# Effects of benign ovarian cyst volume and laterality on AMH and CA-125 levels

**DOI:** 10.1186/s12905-025-03842-9

**Published:** 2025-07-04

**Authors:** Aykut Kından, Aziz Kından, Ahmet Kurt, Volkan Özgür Akbulut, Berna Dilbaz

**Affiliations:** 1 Department of Obstetrics and Gynecology, Pursaklar State Hospital, Mimar Sinan Mh. Çağatay Sk. No:39 , Pursaklar / ANKARA, 06145 Turkey; 2 Department of Perinatology, Etlik City Hospital, Ankara, Turkey; 3 Department of Obstetrics and Gynecology, Çaldıran State Hospital, Van, Turkey

**Keywords:** Ovarian cysts, anti-Müllerian hormone, CA-125 antigen, Endometrioma, Ovariy/surgery, Ovarian reserve, Bilateral ovarian cysts

## Abstract

**Objective:**

This study aimed to investigate the effects of cyst volume and bilaterality on serum Anti-Müllerian Hormone (AMH) and CA-125 levels in patients with benign ovarian cysts.

**Methods:**

We retrospectively analyzed 139 patients who underwent cystectomy for benign ovarian cysts. Serum AMH and CA-125 levels were measured preoperatively and at the second post-operative month.

**Results:**

The mean preoperative AMH level was 3.96 ± 3.48 ng/mL, which significantly decreased to 3.59 ± 3.92 ng/mL postoperatively (*p* = 0.026), reflecting an average decline of 9.3%. CA-125 levels significantly decreased from 53.1 ± 157.2 to 21.0 ± 18.4 U/mL (*p* = 0.002). Patients with bilateral cysts had lower AMH levels at the second month (1.67 ± 0.6 vs. 3.80 ± 4.1 ng/mL; *p* = 0.003) despite similar baseline values.

**Conclusion:**

Bilaterality and cyst volume may negatively affect ovarian reserve postoperatively. The decline in AMH levels, particularly following bilateral ovarian cystectomy, has a significant impact on the ovarian reserve and may have implications for future fertility. Early fertility counselling and ovarian preservation strategies should be considered during surgical planning.

## Introduction

Ovarian cysts are among the most commonly encountered benign pathologies of the female reproductive system [1]. Although these cysts are generally asymptomatic, they may lead to clinical problems such as hormonal imbalance, pelvic pain, and infertility [2, 3]. In some cases, obstetric emergencies may occur because of cyst rupture or ovarian torsion. The volume and location of ovarian cysts can be decisive in the clinical course and treatment approach [4]. In addition, biochemical markers play a significant role in the diagnosis and management of these pathologies. Cancer Antigen 125 (CA-125) is widely used in the preoperative differentiation of benign from malignant cysts;, however Ca-125 may also be elevated in benign ovarian cysts [5]. This can create challenges for the differential diagnosis of benign and malignant ovarian cysts. Anti-Müllerian Hormone (AMH) is an important biomarker in reproductive endocrinology and is used for evaluation of ovarian reserve [6]. AMH levels can assist in evaluating ovarian function in individuals with ovarian cysts pre and postoperatively [7].

The volume and bilaterality of ovarian cysts are factors that can influence their biological behavior and serum biomarker levels [8]. In this context, understanding the relationship between the clinical characteristics of benign ovarian cysts and biomarker levels may lead to improved diagnostic and management approaches.

Despite the widespread use of serum AMH and CA-125 levels in clinical practice, few studies have evaluated dynamic changes in relation to the volume and bilaterality of benign ovarian cysts. Clarifying these associations is crucial for optimizing surgical planning, predicting postoperative ovarian reserve, and counselling patients regarding fertility outcomes. This study aimed to fill this gap by analyzing the impact of cyst characteristics, including volume and laterality, on these biomarkers in a well-defined patient cohort.

## Materials and methods

This study retrospectively examined the relationship between volume, bilaterality, and serum biomarker levels (AMH and CA-125) of pathologically proven benign ovarian cysts. Data were obtained from the electronic records of patients who underwent surgical management of ovarian cystectomy at a tertiary health care center between 2014 and 2020. Ethical approval for this study was obtained from the Ethics Committee of the Etlik Zübeyde Hanım Women’s Health Training and Research Hospital, Ministry of Health, Republic of Turkey, with the decision dated November 29, 2024, and numbered 13. This study was conducted in accordance with all ethical principles and in compliance with the Declaration of Helsinki. The requirement for informed consent was waived owing to the retrospective nature of the study and the use of anonymized data.

### Inclusion and exclusion criteria

The inclusion criteria were as follows: patients diagnosed with ovarian cysts who had undergone surgery for ovarian cystectomy and had received a benign histopathological report. Patients with complete and traceable electronic clinical records who had preoperative measurement of serum AMH and CA-125 levels and preoperative vaginal ultrasonographic measurement of ovarian cyst dimensions were included. Exclusion criteria were the absence of preoperative serum AMH or CA-125 measurements, pregnancy, incomplete or inaccurate clinical records, presence of a malignant pathological diagnosis, or an extragenital pathology. Patients with a history of hormonal therapy within the previous 3 months were excluded to minimize potential effects on AMH or CA-125 levels. Patients who were menopausal or had undergone chemotherapy/radiotherapy were also excluded from the study.

### Study population

The study cohort consisted of consecutive symptomatic patients (pelvic pain, dysmenorrhea, dyspareunia) who underwent surgical intervention for presumed benign ovarian cysts. Patients were comprehensively evaluated preoperatively, and reproductive outcomes were monitored for two months postoperatively. Pregnancy was evaluated one year after the operation.

### Clinical evaluation

Ovarian cysts were assessed preoperatively using transvaginal ultrasonography (TVUS). Ultrasound examination was performed with a 5–9 MHz transvaginal transducer to document volume (measured in centimeters) and bilaterality (classified as unilateral or bilateral). The patients were subsequently categorized into two groups based on histopathological findings: endometriomas and non-endometriotic benign ovarian cysts. Endometriomas were diagnosed preoperatively using transvaginal ultrasound based on the presence of unilocular cysts with homogeneous low-level internal echoes (“ground glass” appearance) and confirmed intraoperatively by visual inspection. Serum AMH levels were measured using a second-generation ELISA kit (Beckman Coulter, USA), with all assays performed in the same laboratory using standardized procedures. The two-month post-operative time point was selected based on prior studies that reported early alterations in AMH and CA-125 levels within this interval, allowing for early postoperative assessment while minimizing the effects of delayed hormonal fluctuations. All surgeries were performed via laparoscopy or laparotomy, depending on the cyst characteristics and surgeon’s preference. All the patients underwent cyst enucleation (complete cyst wall excision) without drainage, puncture, alcoholization, or laser ablation. Hemostasis was achieved by bipolar coagulation. Patients were stratified based on cyst laterality (unilateral vs. bilateral) to allow for subgroup comparisons of postoperative changes in AMH levels. Pregnancy outcomes were assessed through prospective follow-up and spontaneous conception was monitored without the use of assisted reproductive techniques. Although AMH is not a direct marker of natural fertility, this study focused primarily on the postoperative ovarian reserve. Other fertility-related variables, such as tubal patency or male sex, were not specifically excluded.

### Cyst volume calculation

The volume of ovarian cysts was calculated using the prolate ellipsoid formula, which is commonly applied in medical imaging to estimate the volume of three-dimensional structures. According to this method, cyst volume (cm³) was determined as 0.5233× anteroposterior diameter (cm)×transverse diameter(cm)×longitudinal diameter [9]. Preoperative measurements were obtained using TVUS with a 5–9 MHz transvaginal probe, and three orthogonal diameters (anteroposterior, transverse, and longitudinal) were recorded for each cyst. The total ovarian volume was calculated by summing the cyst volume and the remaining healthy ovarian tissue. Preoperative ovarian tissue volume was determined by subtracting the cyst volume from the total ovarian volume. This standardized approach ensures reproducible volumetric assessment, facilitating accurate evaluation of the relationship between cyst characteristics and serum biomarker levels.

### Statistical analysis

Statistical analyses were performed using SPSS software (version 27.0; IBM Corp., Armonk, NY, USA). The normality of the data distribution was assessed using the Kolmogorov-Smirnov test. For comparisons between groups, Student’s t-test or Mann-Whitney U test was applied for continuous variables, depending on the normality of the data. Chi-square or Fisher’s exact test was used for categorical variables. Paired comparisons of preoperative and postoperative parameters were conducted using the paired t-test or Wilcoxon signed-rank test as appropriate. Correlations between continuous variables were evaluated using Pearson’s or Spearman’s correlation coefficient based on data distribution. All statistical tests were two-tailed, and a p-value of less than 0.05 was considered statistically significant. An a priori power analysis was performed using G*Power (version 3.1). A minimum sample size of 130 patients was calculated to detect a medium effect size (Cohen’s d = 0.5) with 80% power and α = 0.05 in a two-tailed test.

## Results

In total, 139 patients were included in this study. Of these, 112 had histologically confirmed non-endometriotic benign ovarian cysts and 27 were diagnosed with endometriomas. The most common non-endometriotic subtypes were serous cystadenomas (*n* = 25), mucinous cysts (*n* = 21), mature cystic teratomas (*n* = 20), follicular cysts (*n* = 17), and corpus luteum cysts (*n* = 7).

The mean preoperative serum AMH level was 3.96 ± 3.48 ng/mL, which slightly decreased to 3.59 ± 3.92 ng/mL in the second postoperative month. The mean difference was 0.37 ng/mL, with a 95% confidence interval (CI) of -0.50 to 1.24. The mean pre-operative CA-125 level was 53.1 ± 157.2 U/mL, which significantly declined to 21.0 ± 18.4 U/mL postoperatively. The mean difference was 32.1 U/mL, with a 95% CI of 5.79 to 58.41 (Table-1).

**Table 1:** Demographic and clinical characteristics, pre and post-operative serum AMH and CA-125 levels of the patient group

	Mean±S.D.
**Age (years)**	28.2±11.2
**Gravida (n)**	0.6±1.4
**Parity (n)**	1.4±1.3
**Number of living children (n)**	1.4±1.3
**Total Cyst Volume (cm** ^**3**^ **)**	315.2±654.2
**AMH Preoperative (ng/mL)**	3.96±3.48
**AMH at 2nd Month (ng/mL)**	3.59±3.92
**CA-125 (U/mL)**	53.1±157.2
**CA-125 at 2nd Month (U/mL)**	21.0±18.4

The most commonly employed surgical technique was laparoscopy (72.7%), while laparotomy was performed in 27.3% of the cases. In terms of cyst localization, 53.2% of the cysts were located on the left side, 37.4% on the right side, and 9.4% on both sides. Douglas obliteration was observed in 15.8% of the patients (Table 2). A history of previous pregnancy was noted in 11.9% of patients with unilateral cysts and 7.7% of those with bilateral cysts before the operation, and this difference was not statistically significant (*p* = 0.650) (Table 2).

**Table 2:** Comparison of surgical characteristics, intraoperative findings, and pre-operative reproductive history between patients with unilateral and bilateral cysts

		Unilateral (*n* = 126)	Bilateral (*n* = 13)	
		Count (%)	Count (%)	*p* value
**Surgery Type**	Laparoscopy	91 (72.2)	10 (76.9)	0.717
	Laparotomy	35 (27.8)	3 (23.1)	
**Douglas Obliteration**		21 (16.7)	1 (7.7)	0.399
**Pre-operative pregnancy**		15 (11.9)	1 (7.7)	0.650

Laparoscopy was the most frequently performed surgical procedure in both groups, with rates of 72.2% in unilateral cases and 76.9% in bilateral cases (*p* = 0.717). Douglas obliteration was observed in 16.7% of patients with unilateral cysts and 7.7% of those with bilateral cysts, with no statistically significant difference between the groups (*p* = 0.399).

Following cystectomy, pregnancy occurred in 11.5% (*n* = 16) of patients, while 88.5% (*n* = 123) did not conceive. The abortion rate was 2.2% (*n* = 3). Term pregnancy was observed in 7.2% (*n* = 10) of cases, whereas preterm birth occurred in 1.4% (*n* = 2). The incidence of stillbirth was 0.7% (*n* = 1).

Cyst volume was larger in the bilateral cyst group [median: 111.0 cm³ (IQR: 84.0–511.0)] compared to the unilateral group [105.0 cm³ (IQR: 36.0–294.0)]; however, this difference was not statistically significant (*p* = 0.366). Preoperative serum AMH levels were similar between the two groups (unilateral: 4.01 ± 3.4 ng/mL; bilateral: 3.40 ± 4.1 ng/mL; *p* = 0.615). However, at the second post-operative month, serum AMH levels were significantly lower in the bilateral group (unilateral: 3.80 ± 4.1 ng/mL; bilateral: 1.67 ± 0.6 ng/mL; *p* = 0.003), with a mean difference of 2.13 ng/mL (95% CI: 1.34 to 2.92). Pre-operative serum CA-125 levels were higher in the bilateral cyst group [median: 24.5 U/mL (IQR: 14.0–54.0)] than in the unilateral group [16.0 U/mL (IQR: 10.0–30.0)], although this difference was not statistically significant (*p* = 0.147). At the second post-operative month, no statistically significant difference in CA-125 levels was observed between the groups [unilateral: 16.0 U/mL (IQR: 10.0–22.0); bilateral: 17.5 U/mL (IQR: 10.0–25.0); *p* = 0.506], with a mean difference of -9.90 U/mL (95% CI: -29.25 to 9.45) (Table 3).

**Table 3:** Comparison of cyst volume and serum AMH and CA-125 levels between unilateral and bilateral benign ovarian cysts

	Unilateral (*n* = 126)	Bilateral (*n* = 13)	*p* value
**Total Cyst Volume (cm** ^**3**^ **)**	105.0 (36.0; 294.0)	111.0 (84.0; 511.0)	0.366 ^b^
**AMH Preoperative (ng/mL)**	4.01±3.4	3.40±4.1	0.615^a^
**AMH at 2nd Month (ng/mL)**	3.80±4.1	1.67±0.6	**0.003** ^**a**^
**CA-125 (U/mL)**	16.0 (10.0; 30.0)	24.5 (14.0; 54.0)	0.147 ^b^
**CA-125 at 2nd Month (U/mL)**	16.0 (10.0; 22.0)	17.5 (10.0; 25.0)	0.506 ^b^

Continuous variables are presented as median (25th–75th percentile) or mean ± standard deviation (SD), depending on the data distribution. Statistically significant p-values are highlighted in bold. Superscripts indicate the statistical tests applied: “a” refers to the independent samples t-test, and “b” refers to the Mann-Whitney U test.

A statistically significant difference was observed in total cyst Volume, with endometriomas being larger than non-endometriotic cysts (mean difference: 333.7 cm³; 95% CI: -107.97 to 775.37; *p* = 0.017). Preoperative CA-125 levels were significantly higher in the endometrioma group than in the non-endometriotic cyst group (mean difference: 62.6 U/mL; 95% CI: 8.09 to 117.11; *p* = 0.038). Similarly, CA-125 levels in the second postoperative month were markedly elevated in patients with endometriomas (mean difference: 21.3 U/mL; 95% CI: 11.30, 31.30; *p* < 0.001) (Table 4).

**Table 4:** Comparison of endometriomas with non-endometriotic cysts in terms of cyst volume and serum AMH and CA-125 levels

	Non-endometriotic Cysts (*n* = 112)	Endometrioma (*n* = 27)	
	Mean±S.D.	Mean±S.D.	*p* value
**Total Cyst Volume (cm** ^**3**^ **)**	250.4±447.8	584.1±1150.1	**0.017** ^**b**^
**AMH Preoperative (ng/mL)**	3.91±3.72	4.15±2.43	0.779 ^a^
**AMH at 2nd Month (ng/mL)**	3.82±4.63	3.14±1.98	0.569 ^a^
**CA-125 (U/mL)**	41.4±162.8	104.0±120.4	**0.038** ^**b**^
**CA-125 at 2nd Month (U/mL)**	15.2±9.6	36.5±26.1	**< 0.001** ^**b**^

Statistically significant p-values are highlighted in bold. Superscripts indicate the statistical tests applied: “a” refers to the independent samples t-test, and “b” refers to the Mann-Whitney U test.

A positive correlation was observed between the total cyst volume and preoperative CA-125 levels; however, this relationship was not statistically significant (*r* = 0.024, *p* = 0.785). Similarly, no statistically significant correlation was found between cyst volume and CA-125 levels in the second postoperative month (*r* = 0.048, *p* = 0.676). A negative correlation was noted between preoperative serum AMH levels and total cyst volume; however, this relationship was not statistically significant (*r*=-0.044, *p* = 0.677). Similarly, no significant correlation was found between serum AMH levels and total cyst volume in the second postoperative month (*r*=-0.085, *p* = 0.576) (Table 5).

**Table 5:** Correlation between total cyst volume and pre and post-operative serum CA-125 and AMH levels

		CA-125	CA-125 at 2nd Month
**Total Cyst Volume (cm** ^**3**^ **)**	Pearson Correlation	0.024	0.048
	p value	0.785	0.676
**AMH Preoperative (ng/mL)**	Pearson Correlation	-0.044	-0.009
	p value	0.677	0.943
**AMH at 2nd Month (ng/mL)**	Pearson Correlation	-0.085	0.044
	p value	0.576	0.804

## Discussion

The ovarian reserve can be assessed using several markers including serum AMH, antral follicle count (AFC) via transvaginal ultrasound, and follicle-stimulating hormone (FSH) levels [10]. Among these, AMH is considered to be one of the most reliable because of its cycle-independent stability. Evaluation of patients with benign ovarian cysts revealed that serum AMH levels and cyst volume are critical variables, both preoperatively and postoperatively. Notably, in the second postoperative month, a significant decline in serum AMH levels was observed in the bilateral cyst group. This suggests that the removal of bilateral cysts may lead to a larger loss of normal ovarian tissue and thus have a more pronounced negative impact on ovarian reserve. The decline in AMH levels after cystectomy is mainly due to the inadvertent loss of healthy ovarian tissue and follicles during excision, especially in endometriomas, where cyst walls adhere tightly to the ovarian cortex. Additionally, thermal damage from electrocautery used for hemostasis may further reduce follicular density, leading to a measurable decline in the ovarian reserve [11, 12]. Additionally, while the effect of total cyst volume on serum CA-125 levels was not statistically significant, bilateral cysts had higher CA-125 levels. These findings highlight the importance of considering cyst localization and volume in the surgical management of benign ovarian cysts and counselling patients preoperatively about possible postoperative changes in ovarian reserve. These results provide valuable insights into the impact of surgical interventions on ovarian reserve and reproductive potential, emphasizing the need for tailored clinical approaches [13, 14].

Early diagnosis of ovarian malignancy remains a significant challenge, as neither biomarkers nor imaging techniques alone are sufficient for definite diagnosis prior to surgery. Consequently, various studies have focused on developing algorithms that integrate serial radiological assessments, biomarker measurements, or a combination of both [15–17]. Olivier et al. demonstrated that the combination of CA-125 with transvaginal ultrasound (TVUS) yielded a positive predictive value of 40% for ovarian cancer; however, this approach proved more effective for detecting advanced-stage disease [17]. Similarly, Timmerman et al. (2010) reported that logistic regression models based on ultrasound imaging data achieved high accuracy in distinguishing between benign and malignant adnexal masses.

Sharma et al. (2020) investigated the relationship between benign ovarian cysts and CA-125 levels, concluding that there was no statistically significant correlation between total cyst volume and CA-125 levels [5]. Our findings are consistent with those of Sharma et al., who found no significant impact of cyst volume on CA-125 levels. However, our study also revealed that bilateral cysts demonstrated significantly reduced serum AMH levels in the second postoperative month compared with unilateral cysts. Compared to other benign ovarian cysts, endometriomas are associated with a more significant decline in the ovarian reserve. This is attributed to their inflammatory nature and close adherence to ovarian tissue, leading to the inadvertent removal of healthy follicles during surgical excision. A systematic review and meta-analysis by Muzii et al. (2018) demonstrated that patients with unoperated endometriomas had significantly lower AMH levels compared to those with non-endometriotic benign ovarian cysts (mean difference: -0.85 ng/mL; 95% CI: -1.37 to -0.32) [18].

In the present study, serum AMH levels were measured preoperatively and in the second postoperative month. Unlike some studies suggesting partial postoperative recovery of AMH levels, we observed no improvement in the second month. Some studies have reported partial recovery of AMH levels within 3–6 months postoperatively, particularly after unilateral cystectomy [19, 20]. This transient increase is thought to result from improved ovarian perfusion or compensatory follicular activation. However, in bilateral cases, recovery is often limited or absent, reflecting more extensive follicular loss [21]. The levels remained significantly low, indicating a more pronounced and potentially lasting impact on ovarian reserve. This lack of recovery highlights the critical need for careful consideration during the surgical management of bilateral ovarian cysts. The finding that the bilateral cyst group had significantly lower AMH levels in the second postoperative month (mean: 1.67 ng/mL) has important clinical implications. AMH levels < 2 ng/mL are generally considered indicative of diminished ovarian reserve, particularly in women of reproductive age. Therefore, patients with bilateral cysts may be at a higher risk of subfertility or a reduced response to assisted reproductive treatments following surgery. These results underscore the need for individualized preoperative counselling and fertility-preserving surgical techniques for women undergoing bilateral ovarian cystectomy. This apparent discrepancy with previous reports of AMH recovery may be attributed to the shorter follow-up period in our study, predominance of bilateral cyst cases, and differences in surgical hemostasis techniques, all of which may influence the extent and pace of AMH normalization. Several mechanisms have been proposed to explain the recovery of serum AMH levels after surgery, and the restoration of ovarian blood flow may facilitate the release of AMH from the remaining follicular pool as the ovarian vasculature is reestablished and hyperactivation of granulosa cells of the remaining follicles as a compensatory response to ovarian damage, thus potentially increasing AMH secretion per follicle despite a reduction in the total follicle count. Some researchers have suggested that atretic follicles might be rescued during the recovery period, as demonstrated in hemicastrated rats, which showed an increased number of ovulating follicles. These additional follicles may originate from a cohort of smaller healthy follicles termed “reserve follicles” [22]. Finally, the most debated theory is that surgery-induced inflammation might promote ovarian follicle regeneration, either from the ovarian surface epithelium [23, 24] or bone marrow-derived stem cells [25]. The present study demonstrated a significant reduction in serum AMH levels following surgical intervention, which was attributed to surgery-related ovarian damage. Additionally, AMH levels showed partial recovery over time, reaching approximately 65% of preoperative levels within 3 months. The study highlighted that AMH decline was more pronounced in cases of bilateral cystectomy than in those of unilateral cystectomy. Although we did not specifically analyze AMH decline according to surgical technique (laparoscopy vs. laparotomy), our findings regarding bilateral cysts align with the literature indicating greater ovarian reserve impairment in such cases [26]. Furthermore, our results emphasize the more pronounced negative impact of bilateral cysts on ovarian reserve, underlining the need to consider this factor when preserving ovarian reserve.

Recent studies have underscored the significant impact of endometrioma characteristics, particularly bilaterality and size, on the ovarian reserve. Karada et al. demonstrated that patients with bilateral endometriomas exhibited significantly lower serum AMH levels than those with unilateral cysts, highlighting the detrimental effect of bilaterality on ovarian reserve [27]. Furthermore, a systematic review and meta-analysis by Younis and Taylor confirmed that surgical excision of endometriomas leads to a significant reduction in serum AMH levels, with the decline being more pronounced in cases involving bilateral cysts [28]. These findings align with our study, which observed a substantial decrease in postoperative AMH levels in patients with bilateral cysts, emphasizing the need for careful preoperative counselling and consideration of fertility-preserving strategies.

Bilateral ovarian cystectomy is associated with a greater risk of ovarian tissue damage owing to the cumulative effects of surgical intervention on both ovaries. The need for bilateral excision increases the likelihood of inadvertent removal of healthy follicles, especially in cysts with adherent walls such as endometriomas [29]. Furthermore, thermal coagulation for hemostasis may be applied more extensively, leading to greater tissue necrosis and follicular loss [30]. Additionally, bilateral procedures may transiently impair ovarian perfusion, contributing to ischemia-reperfusion injury and subsequent oxidative stress, which can further compromise the follicular reserve [31].

This study has several strengths. It evaluates both AMH and CA-125 levels pre- and post-operatively in a well-defined cohort of patients undergoing ovarian cystectomy. The inclusion of both unilateral and bilateral cysts allowed subgroup comparisons based on cyst characteristics. Additionally, all biomarker assessments were performed in the same laboratory using standardized protocols, minimizing inter-assay variability. However, this study has several limitations. First, the retrospective design may have introduced selection bias, as the data were collected from medical records. Second, the relatively small number of patients with bilateral cysts (*n* = 13) may have reduced the statistical power and limited the generalizability of the subgroup findings. Third, biomarker levels were assessed only at baseline and the second postoperative month, which may not reflect long-term trends. Furthermore, the study did not control for potential confounders such as preoperative hormonal therapy. In addition, the single-center design may restrict the generalizability of the findings to a broader population. Finally, the absence of a non-surgical control group limits the ability to attribute the observed biomarker changes exclusively to surgical intervention.

These findings emphasize the importance of considering cyst volume and localization in the surgical management of benign ovarian cysts, particularly highlighting the potential impact of bilateral cysts on the ovarian reserve. Additionally, the prolonged recovery of serum AMH levels following surgical intervention should be considered during individualized patient management planning. In this context, our results may assist clinicians in refining their surgical decision making and providing fertility counselling tailored to cyst characteristics.

## Data Availability

The data used and analyzed in this study are available from the corresponding author upon reasonable request. Due to privacy, ethical considerations, or contractual obligations, certain restrictions may apply. Where applicable, publicly available datasets and sources have been cited in the manuscript. Any additional supporting information can be shared upon request in accordance with institutional and funding body guidelines.

## Abbreviations

AMH: Anti-Müllerian Hormone
CA-125: Cancer Antigen 125
TVUS: Transvaginal Ultrasonography
ELISA: Enzyme-Linked Immunosorbent Assay
AFC: Antral Follicle Count
FSH: Follicle-Stimulating Hormone
SD: Standard Deviation
IQR: Interquartile Range
IRB: Institutional Review Board
